# The 12p13.33/*RAD52* Locus and Genetic Susceptibility to Squamous Cell Cancers of Upper Aerodigestive Tract

**DOI:** 10.1371/journal.pone.0117639

**Published:** 2015-03-20

**Authors:** Manon Delahaye-Sourdeix, Javier Oliver, Maria N. Timofeeva, Valérie Gaborieau, Mattias Johansson, Amélie Chabrier, Magdalena B. Wozniak, Darren R. Brenner, Maxime P. Vallée, Devasena Anantharaman, Pagona Lagiou, Ivana Holcátová, Lorenzo Richiardi, Kristina Kjaerheim, Antonio Agudo, Xavier Castellsagué, Tatiana V. Macfarlane, Luigi Barzan, Cristina Canova, Nalin S. Thakker, David I. Conway, Ariana Znaor, Claire M. Healy, Wolfgang Ahrens, David Zaridze, Neonilia Szeszenia-Dabrowska, Jolanta Lissowska, Eleonora Fabianova, Ioan Nicolae Mates, Vladimir Bencko, Lenka Foretova, Vladimir Janout, Maria Paula Curado, Sergio Koifman, Ana Menezes, Victor Wünsch-Filho, José Eluf-Neto, Paolo Boffetta, Leticia Fernández Garrote, Diego Serraino, Marcin Lener, Ewa Jaworowska, Jan Lubiński, Stefania Boccia, Thangarajan Rajkumar, Tanuja A. Samant, Manoj B. Mahimkar, Keitaro Matsuo, Silvia Franceschi, Graham Byrnes, Paul Brennan, James D. McKay

**Affiliations:** 1 Genetic Cancer Susceptibility group (GCS), International Agency for Research on Cancer (IARC), Lyon, France; 2 Genetic Epidemiology group (GEP), International Agency for Research on Cancer (IARC), Lyon, France; 3 Colon Cancer Genetics Group, Institute of Genetics and Molecular Medicine, University of Edinburgh and Medical Research Council (MRC) Human Genetics Unit, Edinburgh, United Kingdom; 4 Department of Hygiene, Epidemiology and Medical Statistics, University of Athens School of Medicine, Athens, Greece; 5 Institute of Hygiene and Epidemiology, 1st Faculty of Medicine, Charles University, Prague, Czech Republic; 6 University of Turin, Department of Medical Sciences, Unit of Cancer Epidemiology, Turin, Italy; 7 Cancer Registry of Norway, Oslo, Norway; 8 Catalan Institute of Oncology-ICO, IDIBELL. L'Hospitalet de Llobregat, Barcelona, Spain; 9 CIBER Epidemiología y Salud Pública (CIBERESP), Madrid, Spain; 10 School of Medicine and Dentistry, University of Aberdeen, Aberdeen, United Kingdom; 11 General Hospital of Pordenone, Pordenone, Italy; 12 Department of Environmental Medicine and Public Health, University of Padova, Padova, Italy; 13 MRC-HPA Centre for Environment and Health, Respiratory Epidemiology and Public Health, National Heart and Lung Institute, Imperial College, London, United Kingdom; 14 University of Manchester, School of Dentistry, Manchester, United Kingdom; 15 University of Glasgow Dental School, Glasgow, Scotland, United Kingdom; 16 Croatian National Cancer Registry, Croatian National Institute of Public Health, Zagreb, Croatia; 17 Trinity College School of Dental Science, Dublin, Ireland; 18 Leibniz Institute for Prevention Research and Epidemiology—BIPS, Bremen, Germany; 19 Faculty of Mathematics and Computer Science, University of Bremen, Bremen, Germany; 20 Institute of Carcinogenesis, Cancer Research Centre, Moscow, Russian Federation; 21 Department of Epidemiology, Institute of Occupational Medicine, Lodz, Poland; 22 Department of Cancer Epidemiology and Prevention, M. Sklodowska-Curie Memorial Cancer Center and Institute of Oncology, Warsaw, Poland; 23 Regional Authority of Public Health, Banska Bystrica, Slovakia; 24 Saint Mary General and Esophageal Surgery Clinic, Carol Davila University of Medicine and Pharmacy, Bucharest, Romania; 25 Department of Cancer Epidemiology and Genetics, Masaryk Memorial Cancer Institute and Masaryk University, Brno, Czech Republic; 26 Palacky University, Olomouc, Czech Republic; 27 International Prevention Research Institute (IPRI), Ecully, France; 28 National School of Public Health/FIOCRUZ, Rio de Janeiro, Brazil; 29 Universidade Federal de Pelotas, Pelotas, Brazil; 30 Universidade de Sao Paulo, Sao Paulo, Brazil; 31 The Tisch Cancer Institute Mount Sinai School of Medicine, New York, NY, United States of America; 32 Institute of Oncology and Radiobiology, Havana, Cuba; 33 Centro di Riferimento Oncologico, IRCSS, Unit of Epidemiology and Biostatistics, Aviano, Italy; 34 Department of Genetics and Pathology, International Hereditary Cancer Center, Pomeranian Medical University, Szczecin, Poland; 35 Department of Otolaryngology and Laryngological Oncology, Pomeranian Medical University, Szczecin, Poland; 36 Institute of Public Health, Section of Hygiene, Faculty of Medicine, Università Cattolica del Sacro Cuore, Rome, Italy; 37 Dept. of Molecular Oncology, Cancer Institute (WIA), Chennai, Tamil Nadu, India; 38 Cancer Research Institute, Advanced Centre for Treatment, Research and Education in Cancer, Tata Memorial Centre, Navi Mumbai, India; 39 Department of Health Promotion, Division of Oral Pathology, Kyushu Dental University, Kitakyushu, Japan; 40 Infections and Cancer Epidemiology group (ICE), International Agency for Research on Cancer (IARC), Lyon, France; 41 Biostatistics group (BST), International Agency for Research on Cancer (IARC), Lyon, France; Ohio State University Medical Center, UNITED STATES

## Abstract

Genetic variants located within the 12p13.33/*RAD52* locus have been associated with lung squamous cell carcinoma (LUSC). Here, within 5,947 UADT cancers and 7,789 controls from 9 different studies, we found rs10849605, a common intronic variant in *RAD52*, to be also associated with upper aerodigestive tract (UADT) squamous cell carcinoma cases (OR = 1.09, 95% CI: 1.04–1.15, p = 6x10^−4^). We additionally identified rs10849605 as a *RAD52 cis*-eQTL inUADT(p = 1x10^−3^) and LUSC (p = 9x10^−4^) tumours, with the UADT/LUSC risk allele correlated with increased *RAD52* expression levels. The 12p13.33 locus, encompassing rs10849605/*RAD52*, was identified as a significant somatic focal copy number amplification in UADT(n = 374, q-value = 0.075) and LUSC (n = 464, q-value = 0.007) tumors and correlated with higher *RAD52* tumor expression levels (p = 6x10^−48^ and p = 3x10^−29^ in UADT and LUSC, respectively). In combination, these results implicate increased *RAD52* expression in both genetic susceptibility and tumorigenesis of UADT and LUSC tumors.

## Introduction

Upper aerodigestive tract (UADT) cancers, comprising of the oral cavity, larynx and esophagus, are the fourth most common cause of cancer death worldwide [1]. While consumption of tobacco and alcohol are the main UADT cancers risk factors [2], genetic susceptibility has been hypothesized to play a role in the pathogenesis of this disease [3,4].

Exposure to tobacco and alcohol leads to cell damage and DNA alterations that, in the absence of appropriate repair, may cause cell cycle deregulation and cancer development [5,6]. Homologous Recombination (HR) is an important manner by which cells repair DNA lesions [7,8]. The *RAD52* gene is involved in the homologous recombination DNA repair process [9] by mediating RAD51, a central HR gene that forms a helical nucleoprotein filament involved in the search for homology and strand pairing [10].

Genome wide association studies (GWAS) have implicated the rs10849605 genetic variant at 12p13.33, the locus that encompasses *RAD52* in the human genome, to be associated with a modest, but statistically significant, increased risk of lung cancer [11,12]. It appears most relevant to lung squamous cell carcinoma (LUSC) and small cell lung cancers, but with little evidence within lung adenocarcinomas (LUAD) [11,12]. Although the molecular mechanisms contributing to initiation and progression are still poorly understood, squamous cell carcinomas (SCC) of different anatomical sites share many phenotypic and molecular characteristics with each other [13]. The aim of the present study was to investigate *RAD52* in the context of genetic susceptibility to SCC of the UADT, to explore how this association might be mediated and examine the somatic mutation events at the *RAD52* 12p13.33 locus.

## Materials and Methods

### Study subjects

A total of 9 case-control studies of UADT cancer participated in our present study totalling 5,947 UADT cancer cases and 7,789 controls. Study designs and population characteristics have been described in more details previously [3,14,15] and are briefly described in Table 1. In most studies, the control subjects were frequency matched to the cases on age, sex, and additional factors (e.g., study site and hospital). Written informed consent was obtained from all study subjects, and the investigations were approved by the institutional review boards at each study center. Analysis was restricted to squamous cell carcinomas.

**Table 1:** Demographic characteristics of the cases and controls included in the genetic susceptibility study of *RAD52*/rs10849605.

	Cases		Controls		OR	95%CI	p-value
**Gender**							
Male	4604	77.4	5750	73.8			
Female	1343	22.6	2039	26.2	0.76	(0.70–0.83)	3.7x10^−10^
**Age group**							
<50 years old	1083	18.2	1564	20.1			
> = 50 years old	4483	75.4	6224	79.9	1.26	(1.14–1.38)	1.6x10^−6^
Missing	381	6.4	1	0.0			
**Smoking status**							
Never smokers	825	13.9	2501	32.1			
Ever smokers	4735	79.6	4309	55.3	4.15	(3.74–4.62)	2.2x10^−154^
Former	1169		1923		2.56	(2.25–2.90)	9.8x10^−48^
Current	3511		2279		6.51	(5.80–7.31)	2.4x10^−218^
Missing	55		107				
Missing	387	6.5	979	12.6			
**Drinking status**							
Never drinkers	904	15.2	1892	24.3			
Ever drinkers	4653	78.2	4918	63.1	2.66	(2.38–2.97)	3.0x10^−65^
Former	758		521		3.71	(3.13–4.41)	3.5x10^−51^
Current	2650		2588		3.13	(2.72–3.60)	1.9x10^−58^
Missing	1245		1809				
Missing	390	6.6	979	12.6			
**Site of tumor**							
Oral/Oropharynx	3105	52.2					
Larynx/Hypopharynx	2182	36.7					
Esophagus	636	10.7						
Missing	24	0.4					

OR, CI and p-values represent the risk of UADT in each substrata, adjusted for sex and study specific country of origin.

### Genotyping

The rs10849605 was genotyped using Illumina bead arrays or TaqMan genotyping (C__1244798_10, Applied Biosystems, Carlsbad, CA) at IARC as described elsewhere [3]. The performance of Taqman assays was validated by re-genotyping samples of known genotype (for example HapMap). The genotype distribution was in accordance with that expected by Hardy-Weinberg equilibrium in each country/study. All subsequent genotyping achieved an internal study duplicate concordance rate of >99%.

### The Cancer Genome Atlas data

We accessed to the Head and Neck Squamous Cell Carcinoma (HNSC), Lung Squamous Cell Carcinoma (LUSC) and Lung Adenocarcinoma (LUAD) components of the TCGA data (TCGA Project Number #3230 and #2731). This data is accessible using the dbGAP via the TGCA (https://tcga-data.nci.nih.gov/tcga/). Data were downloaded either from https://cghub.ucsc.edu/ for exome sequencing or directly from https://tcga-data.nci.nih.gov/tcga/ for the RNA sequencing, methylation and genotype data.

#### Exome sequencing

We accessed TCGA exome sequencing “level 1” (unprocessed) data for 363 HNSC and 459 LUSC TCGA individuals and completed bioinformatics analysis of their sequence data using Picard, GATK, MuTect and Somatic Indel detector (Methods A in S1 File). Subsequently we used in house bioinformatics pipelines (Methods A in S1 File) to determine the highest quality variant calls. Somatic point mutations were exonic functional variants defined as either truncating, impacting splicing or missense variants predicted as deleterious by SIFT/POLYPHEN2 [16,17].

#### Copy Number Variation

Samples were hybridized using the Genome-Wide Human SNP Array 6.0 platform at the Genome Analysis Platform of the Broad Institute. We retrieved level 3 TCGA data of 374 HNSC, 464 LUSC and 476 LUAD individuals containing normalized log_2_ ratios of the fluorescence intensities between the target sample and a reference sample. We only included in our analysis individuals for whom both tumor and corresponding normal calls were available. For a segment, we considered log_2_(ratio) < -0.5 to be an indication of a loss, and a log_2_(ratio) > 0.5 to indicate a gain. Segments with log_2_(ratio) of between −0.5 and 0.5 were not retained as somatic copy number alterations. Annotation was done adding the genes contained in each of the remaining segments using EnsEMBL databases [18].

#### RNA sequencing

RNA sequencing (RNA-seq) TCGA data was retrieved the “level 3” data for 263 HNSC, 223 LUSC and 125 LUAD individuals. Normalization of this data is further detailed within the statistical methods section.

#### Methylation

TCGA methylation data was analysed on the Illumina Infinium HumanMethylation 450K BeadChip assay. We accessed TCGA methylation “level 2” data for 263 HNSC, 223 LUSC and 125 LUAD individuals. We estimated the methylated level of each CpG site by calculating the M-value (log_2_(ratio of methylated and unmethylated probes)) using TCGA level 2 data [19]. Methylation level 2 data is already background-corrected.

#### rs10849605 TCGA genotypes

rs10849605 is located inside the 5’ region of *RAD52* and was not covered by exome sequencing. Therefore we derived the genotypes for 263 HNSC, 223 LUSC and 125 LUAD individuals using the Affymetrix 6.0 SNP array TCGA data.

### Statistical methods

#### Association analysis

The association between the variants and UADT cancer risk was estimated by odds ratio (ORs) and 95% confidence intervals (CIs) per allele under the log-additive model and genotype derived from multivariate unconditional logistic regression, with sex and study specific country of origin included in the model as covariates (S1 Table). Heterogeneity of ORs was assessed using the Cochran’s Q test. Statistical analyses were performed using SAS version 9.3 (SAS Institute, Cary, NC, USA).

To control for potential ethnic heterogeneity between cases and controls, we performed a principal components analysis using the EIGENSTRAT package of the EIGENSOFT 5.0 software [20] using 12,898 markers in low linkage disequilibrium [21]. We used the resulting 12 statistically significant eigen vectors (as defined by the Tracy-Widom statistics) in the sensitivity analysis (Table A in S1 File).

#### eQTL analyses

The association between rs10849605 germline genotype and *RAD52* tumor expression levels (eQTL) was tested on 263 HNSC, 223 LUSC and 125 LUAD using a linear model. It has been repeatedly observed that tumors acquire somatic alterations that can also influence gene expression, particularly copy number changes and DNA methylation [22–24]. Therefore we tested the eQTL effect of rs10849605 on *RAD52* tumor expression using both adjusted and non-adjusted linear models as described in Table B in S1 File. These statistical analyses were performed using R statistical software (The R Foundation for Statistical Computing, http://www.R-project.org).

In order to control for the impact of population heterogeneity, we inferred population structure of the 263 HNSC, 223 LUSC and 125 LUAD TCGA cases with the Structure software [25] using Hapmap release #23 as the reference population [26] and restricted the eQTL analyses to the 215 HNSC, 192 LUSC and 113 LUAD cases predicted to be of European ancestry (CEU>0.8). On these, we further conducted a principal components analysis similar to the GWAS one. The resulting significant eigen vectors (as defined by Tracy-Widom statistics) were used within the eQTL sensitivity analysis (Table C in S1 File).

#### Copy number analysis—GISTIC

We used a publicly available method, called Genomic Identification of Significant Targets in Cancer (GISTIC) [27,28], version 2.0 to find the significantly amplified or deleted regions using TCGA copy number data. The GISTIC algorithm computes p-values for each marker by comparing the score at each locus to a background score distribution generated by random permutation of the marker locations in each sample. Then they correct the p-values for multiple-hypothesis testing using the Benjamini-Hochberg false discovery rate (FDR) method. Therefore the GISTIC scores represent significance levels and are expressed as q-values (significant below 0.25).

#### RNA sequencing normalization

Level 3 RNA sequencing tumor data that we accessed from the TCGA was already normalized to the kilobase per million reads (RPKM) standard which corrects for species length and read depth [29], but not for diversity of the RNA population. To control for this we applied TMM (Trimmed Mean of M-values) normalization [30] to the RPKM data. This possibly involves a loss of statistical efficiency relative to applying TMM to raw data, since the precision weighting in TMM will no longer function. However it should not add any bias and the loss of efficiency will be small if the read density is close to uniform. We used implementations in the EdgeR package of BioConductor [31] and the voom function of the Bioconductor limma package [32]. The normal expression data being available only for a few cases, it was not possible to perform any differential expression analysis.

## Results

### Germline genetic variation rs10849605 and susceptibility to UADT cancers

We genotyped rs10849605 in 5,947 UADT cancer cases and 7,789 control individuals from 9 studies. Frequency of the minor allele of rs10849605 varied somewhat by country, with the risk allele (C) being more prevalent in Europe and Latin America countries compared to Asia (51% and 49% versus 40% respectively).

**Fig 1:**
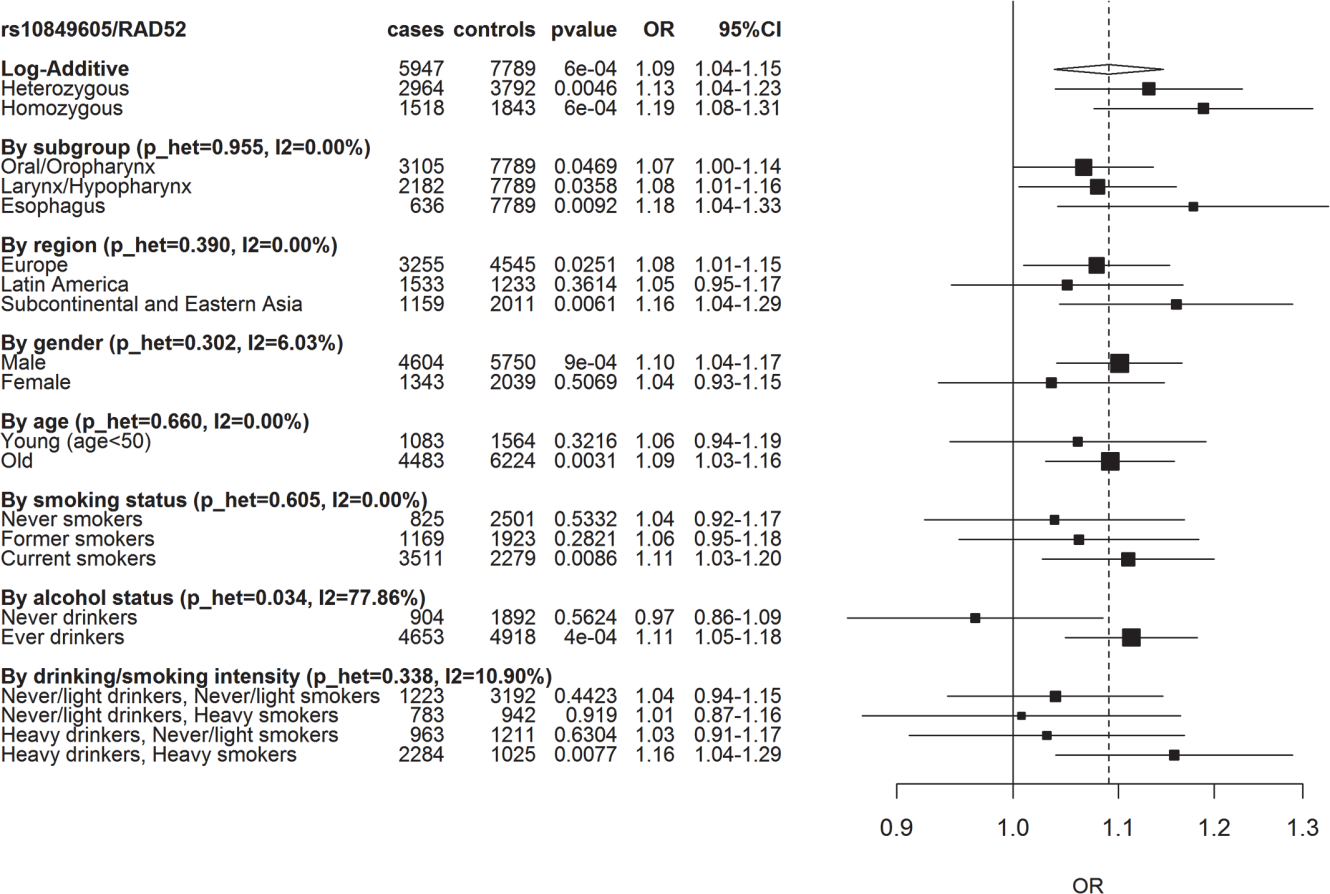
Association between *RAD52* SNP rs10849605 and UADT cancer risk. Squares represent ORs, size of the square represents the inverse of the variance of the log ORs, horizontal lines represent 95% CIs. The solid vertical line indicates OR = 1 and the dashed vertical line the overall OR under the log-additive model. p_het is the p-value for heterogeneity between the different subgroups. I2 is the % of observed variation across subgroups (negative I2 were set to 0).

As observed in squamous cell carcinoma of the lung, the C allele was associated with a modest increase in UADT cancer risk (Fig. 1, p = 6x10^−4^), with the odds ratio (OR) for having one additional risk allele being 1.09 (95%CI: 1.04–1.15). The association appeared relatively consistent across geographic region (Fig. 1), and did not appear sensitive to cryptic population structure within 1,791 cases and 2,531 controls where genome wide data was available to infer genetic ancestry (Table A in S1 File). The association was also consistent within UADT cancer subsites and consumption of tobacco. However, it was more prominent in those that consumed alcohol compared to non-drinkers (p_het 0.03) (Fig. 1). There was little evidence to suggest this variant altered consumption patterns of tobacco and alcohol (p = 0.53 and p = 0.40, respectively, pack/years and ethanol/day taken as a continuous variable).

### Integrated *in-silico* fine mapping of the 12p13.33 locus

We next undertook *in-silico* analysis of the rs10849605 variant and the *RAD52*/12p13.33 locus in the head and neck and lung cancers genomically characterised by the Cancer Genome Atlas (TCGA).

### Expression quantitative trait locus (eQTL) of rs10849605 in HNSC and LUSC

**Fig 2:**
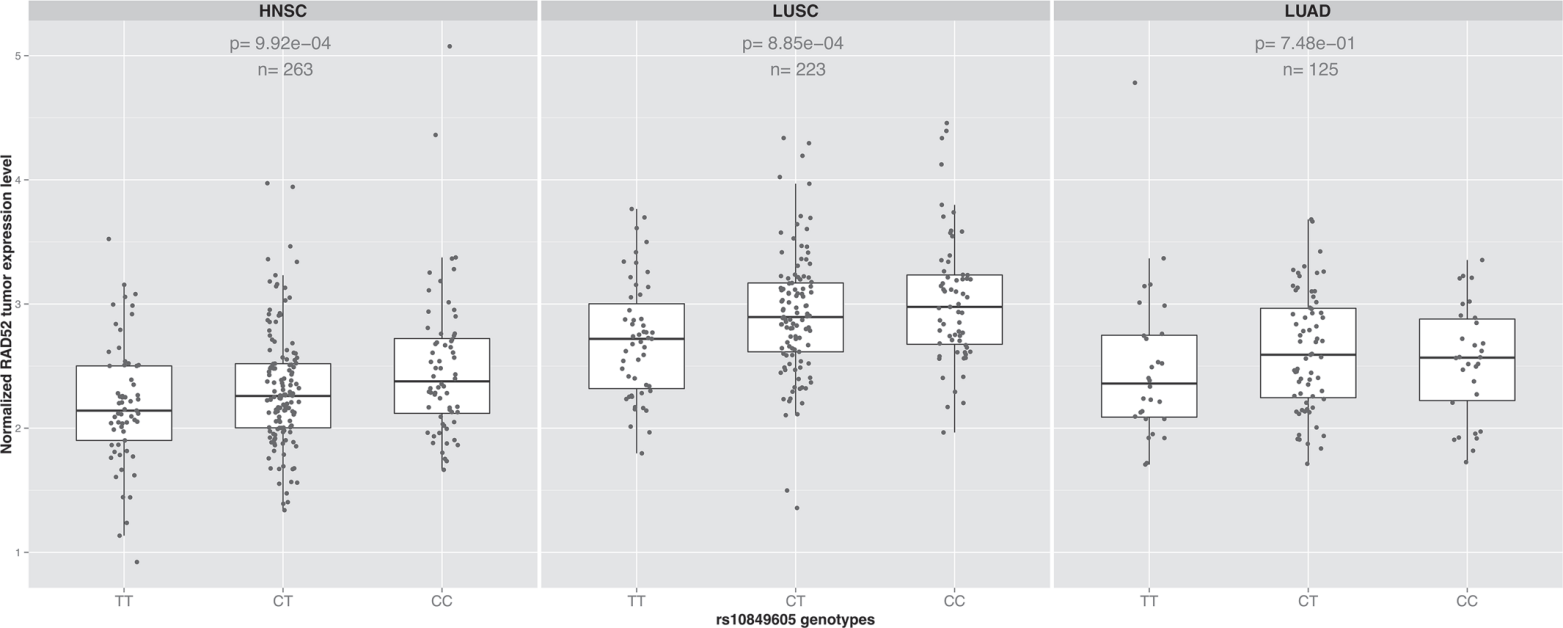
eQTL analysis. Boxplots showing the effect of the genotype for the SNP *RAD52* rs10849605 on *RAD52* tumor expression levels in HNSC, LUSC and LUAD. The risk allele (C) significantly increases *RAD52* expression levels (p = 9x10^−4^ and 8x10^−4^ respectively) in both squamous cancers but not in lung adenocarcinoma (p = 0.75). In contrast, there was no evidence for association between rs10849605 and expression levels of other genes in the 12p13.33 region (Table D in S1 File).

rs10849605 is located near the putative promoter 5’ to the *RAD52* gene, therefore we hypothesized that this, or a correlated proxy variant, might influence *RAD52* gene expression. We performed an expression quantitative trait locus (eQTL) analysis between rs10849605 and *RAD52* expression levels in HNSC (n = 263), using data where both RNAseq of the tumors and genotyping had been carried out by TCGA within the same individuals. rs10849605 was significantly associated with *RAD52* gene expression levels in HNSC (Fig. 2, n = 263, p = 9x10^−4^), suggesting that rs10849605 is a *cis*-eQTL locus for *RAD52*. The C allele of rs10849605, associated with risk of HNSC, was correlated with increased *RAD52* expression levels (Fig. 2). The association was not sensitive either to adjustment for somatic events (copy number or methylation status which may influence eQTL analysis in tumors [22]), HNSC subtype (larynx/hypopharynx, oral cavity, oropharynx) or population structure (Tables B and C in S1 File). A comparable effect was observed in LUSC (Fig. 2, n = 223, p = 8x10^−4^) but no clear eQTL association was observed in lung adenocarcinoma (LUAD, Fig. 2, n = 125, p = 0.75). While statistically significant, the eQTL for rs10849605 accounted for only a small proportion of the variance (approximately 4%) in *RAD52* expression in HNSC and LUSC tumours, an observation in line with the relatively modest genetic risk observed with this variant.

### Somatic alterations at RAD52/12p13.33 in Head and Neck Squamous Cell Carcinoma (HNSC) and LUSC

Within somatic mutations recalled from paired normal-tumor exome sequencing samples of 305 HNSC and 243 LUSC, *RAD52* was rarely mutated somatically (point mutations and insertions deletions), with only 2 HNSC (0.60% of tumors) and one LUSC (0.40% of tumors) patients harbouring a somatic missense variant, and no somatic insertion or deletion observed.

**Fig 3:**
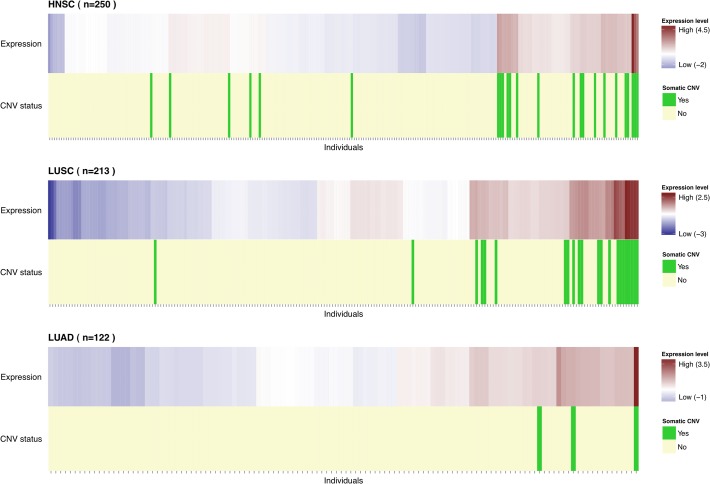
Distribution of individuals by *RAD52* expression. Individuals were ordered by unsupervised clustering based on *RAD52* expression levels. Heatmap represents the scaled RPKM normalized values with higher expression levels represented in red and lower expression levels in blue. The individuals carrying a copy number gain (log_2_(ratio) > 0.5) of *RAD52* are highlighted in green (light yellow otherwise). *RAD52* gain carriers seem to have the same high expression pattern and cluster together. Particularly in LUAD one of the 3 gain carriers has the highest *RAD52* expression level.

By contrast, we analysed the TCGA somatic copy number variation (CNV) data of 374 HNSC, 464 LUSC and 476 LUAD tumors and found that the 12p13.33 locus was a frequent region of copy number gain in HNSC (7.2% of cases) and LUSC (11.2% of cases). Copy number gain of 12p13.33 was observed in a lower proportion of LUAD tumors (3.9% of cases) (Fig. 3). There was a significant difference in the somatic copy number gain frequencies between SCC and LUAD (p = 3x10^−5^). Additionally, we used GISTIC2 statistical program to determine the relative importance of the 12p13.33 gain in comparison with the background rate of copy number changes across the genome [27,28] using the TCGA somatic copy number data. The 12p13.33 region was identified by GISTIC2 as a significant focal amplification in HNSC and LUSC (q-value = 0.075 and 0.007, respectively) but not in LUAD (Figure A in S1 File).

Presence of somatic copy number gain was also correlated with higher *RAD52* expression levels in both HNSC and LUSC tumors, (p = 6x10^−48^ and 3x10^−29^, respectively) (Fig. 3), with copy number at this locus accounting for a large proportion of the variance in *RAD52* tumor expression levels (57% in HNSC and 45% in LUSC). As expected, gene expression levels were correlated with copy number for other genes at 12p13.33 (11 out of 26). However, rs10849605 appeared to influence only *RAD52* expression levels (Table D in S1 File).

## Discussion

Our study has identified rs10849605 to be associated with UADT cancer (p = 6x10^−4^). While the modest nature of this association limited our ability to detect inter-substrata heterogeneity, the association was relatively consistent across the diverse etiological settings of Europe, Japan, Latin America and sub-continental Asia (where tobacco chewing is an important UADT cancer risk factor). We note that differing LD patterns, or cryptic population structure where we were unable to control for, could influence these results. Nevertheless, our findings are consistent with the observation that rs10849605 (or variants correlated with it) have also been associated with lung cancer, and particularly lung squamous cell carcinomas. As found in lung cancer [12], the allele C of the susceptibility variant rs10849605 was associated with a modest increased risk of UADT.

rs10849605 is located at chromosome 12p13.33, a locus that contains the *RAD52* gene. *RAD52* cellular role is DNA double strand break repair via homologous recombination, interacting with multiple DNA repair related genes within this function and therefore a plausible candidate gene to explain this association [33]. Nevertheless, we cannot exclude the possibility of an alternate susceptibility gene to *RAD52* due to linkage disequilibrium. We therefore used an *in-silico* integrative analysis using TCGA expression, genotype and somatic alteration data to fine map this susceptibility locus. 12p13.33 was a region of significant somatic copy number gain in HNSC and LUSC, suggesting that somatic amplification of 12p13.33 is an important molecular event in a subset of tumors. However, the 3MBp amplified region contained multiple genes in addition to *RAD52*. Importantly, rs10849605 was an eQTL in HNSC and LUSC for *RAD52* only, suggesting *RAD52* as the most probable candidate driver gene for both the genetic susceptibility and tumorigenesis at this locus. As an eQTL, the rs10849605 UADT and LUSC risk associated allele (allele C) was correlated with increased *RAD52* expression levels. That higher *RAD52* expression appears involved in both genetic susceptibility and somatic events in UADT and LUSC may indicate that RAD52 activity is enabling tumor cells to have sufficient genome integrity to avoid apoptosis, a trait that may be particularly important within the genotoxic environment created by tobacco smoke and alcohol consumption. Notably, both the eQTL and somatic gains were observed in HNSC and LUSC, but not LUAD, consistent with the lung cancer genetic susceptibility [11,12], reinforcing the importance of this locus in SCC.

A key role of *RAD52* is to provide cells with a level of redundancy in DNA repair [34]. *RAD52* is therefore particularly important in cells deficient in the BRCA1-PALB2-BRCA2 pathway, providing an alternate mechanism for DNA repair [35,36]. Targeted inhibition of *RAD52* in *BRCA2* deficient cells results in genomic instability and cell growth inhibition, leading to the suggestion of *RAD52* as a potential therapeutic target using synthetic lethality approaches [37]. Our results linking *RAD52* higher gene expression to UADT and LUSC, along with our recent observation that a rare truncating *BRCA2* genetic variant, rs11571833 (K3326X) is associated with a 2.5 fold risk of squamous cell carcinomas of the lung and UADT [38,39], suggests that such targeted therapy approaches may be worth investigating in UADT and LUSC tumors.

## Supporting Information

S1 File Methods A. **Figure A, Amplification peaks identified across the genome by GISTIC2 in HNSC, LUSC and LUAD**. The Gistic-scores are shown on the top and the q-values on the bottom. The significance line is drawn at q-value = 0.25 and the significantly amplified locus are annotated on the right side of each plot. The 12p13.33 amplified region is framed and indicated with an arrow. **Table A, Population stratification sensitivity analysis.** Model 1 is the original association analysis logistic regression, adjusted for sex and study specific country of origin. Model 2 further adjusts for population stratification including the 12 significant eigen vectors (as defined by Tracy-Widom statistics) as covariates in the logistic regression. **Table B, eQTL analyses using adjusted and non-adjusted linear models to measure the impact of the rs10849605 genotype on *RAD52* tumor expression levels.** The model measures the effect of the protective allele T for rs10849605. Number of individuals taken into account in the model, beta estimates and p-value are given for the three different cancer types and using the following linear models: 1) Non-adjusted, how the genotype influences the gene expression. 2) For HNSC cancer, the subtype (oral cavity, larynx/hypopharynx or oropharynx) is used as the covariate. 3) *RAD52* somatic copy number is used as the covariate. 4) Since we are interested here in the influence of somatic determinants on an increase of expression and because methylation is inversely correlated with expression (hypermethylated sites tend to decrease expression when hypomethylated sites induce increase in expression), we selected 8 of the 24 CpG sites for being hypomethylated (as defined by a negative M-value across all individuals in all our 3 different cancer sites). Out of these 8, only cg15612927 was significantly associated with expression of *RAD52* in all 3 cancers (p-value < 0.05). Therefore tumor methylation levels of cg15612927 was used as the covariate. 5) The initial model is adjusted for all three somatic alterations (subtype for HNSC, somatic copy number and methylation levels). **Table C, eQTL sensitivity analysis.** The linear model measures the effect of rs10849605 genotype on RAD52 tumor expression levels. The first line presents the results on all TCGA cases we accessed. The second line restricts the analysis on TCGA cases predicted to be of European origin. The last line show the results of the same linear model but adjusted for the statistically significant eigen vectors, as defined by Tracy-Widom (5 for HNSC and LUSC, 8 for LUAD). **Table D, 12p13.33 copy number versus expression and eQTL analysis in HNSC and LUSC**. Association analysis between copy number and expression levels for each given gene in the 12p13.33 region (left side of the table, ‘NA’ if no CNV or expression data available). For the significant associations only, we performed an eQTL analysis to check how rs10849605 genotype influences each given gene expression levels (right side of the table). Significant results are highlighted in green (Bonferroni correction for multiple testing).
(DOCX)

S1 Table Study epidemiological exposures and genetic data. (XLSX)
