# Supplementary material for: The 12p13.33/RAD52 Locus and Genetic Susceptibility to Squamous Cell Cancers of Upper Aerodigestive Tract
Source: PLoS One. 2015 Mar 20;10(3):e0117639. doi: 10.1371/journal.pone.0117639 (PMC4368781; doi:10.1371/journal.pone.0117639)
Supplement: S1 File — Figure A, Amplification peaks identified across the genome by GISTIC2 in HNSC, LUSC and LUAD. The Gistic-scores are shown on the top and the q-values on the bottom. The significance line is drawn at q-value = 0.25 and the significantly amplified locus are annotated on the right side of each plot. The 12p13.33 amplified region is framed and indicated with an arrow. Table A, Population stratification sensitivity analysis. Model 1 is the original association analysis logistic regression, adjusted for sex and study specific country of origin. Model 2 further adjusts for population stratification including the 12 significant eigen vectors (as defined by Tracy-Widom statistics) as covariates in the logistic regression. Table B, eQTL analyses using adjusted and non-adjusted linear models to measure the impact of the rs10849605 genotype on RAD52 tumor expression levels. The model measures the effect of the protective allele T for rs10849605. Number of individuals taken into account in the model, beta estimates and p-value are given for the three different cancer types and using the following linear models: 1) Non-adjusted, how the genotype influences the gene expression. 2) For HNSC cancer, the subtype (oral cavity, larynx/hypopharynx or oropharynx) is used as the covariate. 3) RAD52 somatic copy number is used as the covariate. 4) Since we are interested here in the influence of somatic determinants on an increase of expression and because methylation is inversely correlated with expression (hypermethylated sites tend to decrease expression when hypomethylated sites induce increase in expression), we selected 8 of the 24 CpG sites for being hypomethylated (as defined by a negative M-value across all individuals in all our 3 different cancer sites). Out of these 8, only cg15612927 was significantly associated with expression of RAD52 in all 3 cancers (p-value < 0.05). Therefore tumor methylation levels of cg15612927 was used as the covariate. 5) The initial model is adjuste [file pone.0117639.s001.docx]

**SUPPLEMENTARY DATA**

**File S1.**

1. **Methods A.**
2. **Figure A.** Amplification peaks identified across the genome by GISTIC2 in HNSC, LUSC and LUAD
3. **Table A.** Population stratification sensitivity analysis
4. **Table B**. eQTL analyses using adjusted and non-adjusted linear models to measure the impact of the rs10849605 genotype on *RAD52* tumor expression levels
5. **Table C.** eQTL sensitivity analysis
6. **Table D**. 12p13.33 copy number versus expression and eQTL analysis in HNSC and LUSC

**S1 Table*.*** Study epidemiological exposures and genetic data (Excel sheet).

**Methods A.**

We built a pipeline to analyse exome sequencing data downloaded from the TCGA, which in came in the form of aligned BAM files. We first used Picard MarkDuplicates tool [[1](#_ENREF_1)], followed by a combination of GATK [[2](#_ENREF_2)] and Picard [[1](#_ENREF_1)] algorithms to perform local realignment of the reads around indels and quality score recalibration. We then called germline variants using GATK UnifiedGenotyper, somatic single nucleotide variants with Mutect [[3](#_ENREF_3)] and somatic indels with GATK SomaticIndelDetector [[2](#_ENREF_2)].

Variant calls were annotated using Annovar [[4](#_ENREF_4)] to add information to each called variant such as chromosome, position, gene, type of mutation, known frequencies in public databases, etc.

Subsequently we filtered the variants based on quality (mapping quality, quality of the call, strand bias and PCR duplicates), and we used the NHLBI Exome Sequencing Project (ESP)  [[5](#_ENREF_5)] and the 1000 Genome catalogs [[6](#_ENREF_6)] as well as our own germline catalog to filter out both non-somatic calls present in the tumors and technology specific errors.

Finally we filtered the variants to focus on those that are likely to have an impact on gene function (truncating variants, variants impacting splicing, missense variants that are likely to impact function as predicted by SIFT/POLYPHEN2 [[7](#_ENREF_7),[8](#_ENREF_8)]).

References

1. Picard website. <http://picard.sourceforge.net>

2. DePristo MA, Banks E, Poplin R, Garimella KV, Maguire JR, et al. (2011) A framework for variation discovery and genotyping using next-generation DNA sequencing data. Nat Genet 43: 491-498.

3. Cibulskis K, Lawrence MS, Carter SL, Sivachenko A, Jaffe D, et al. (2013) Sensitive detection of somatic point mutations in impure and heterogeneous cancer samples. Nat Biotechnol 31: 213-219.

4. Wang K, Li M, Hakonarson H (2010) ANNOVAR: functional annotation of genetic variants from high-throughput sequencing data. Nucleic Acids Res 38: e164.

5. Exome Variant Server, NHLBI GO Exome Sequencing Project (ESP), Seattle, WA. URL:. <http://evs.gs.washington.edu/EVS/>

6. Abecasis GR, Auton A, Brooks LD, DePristo MA, Durbin RM, et al. (2012) An integrated map of genetic variation from 1,092 human genomes. Nature 491: 56-65.

7. Ng PC, Henikoff S (2003) SIFT: Predicting amino acid changes that affect protein function. Nucleic Acids Res 31: 3812-3814.

8. Adzhubei IA, Schmidt S, Peshkin L, Ramensky VE, Gerasimova A, et al. (2010) A method and server for predicting damaging missense mutations. Nat Methods 7: 248-249.

**Figure A.** Amplification peaks identified across the genome by GISTIC2 in HNSC, LUSC and LUAD. The Gistic-scores are shown on the top and the q-values on the bottom. The significance line is drawn at q-value=0.25 and the significantly amplified locus are annotated on the right side of each plot. The 12p13.33 amplified region is framed and indicated with an arrow.


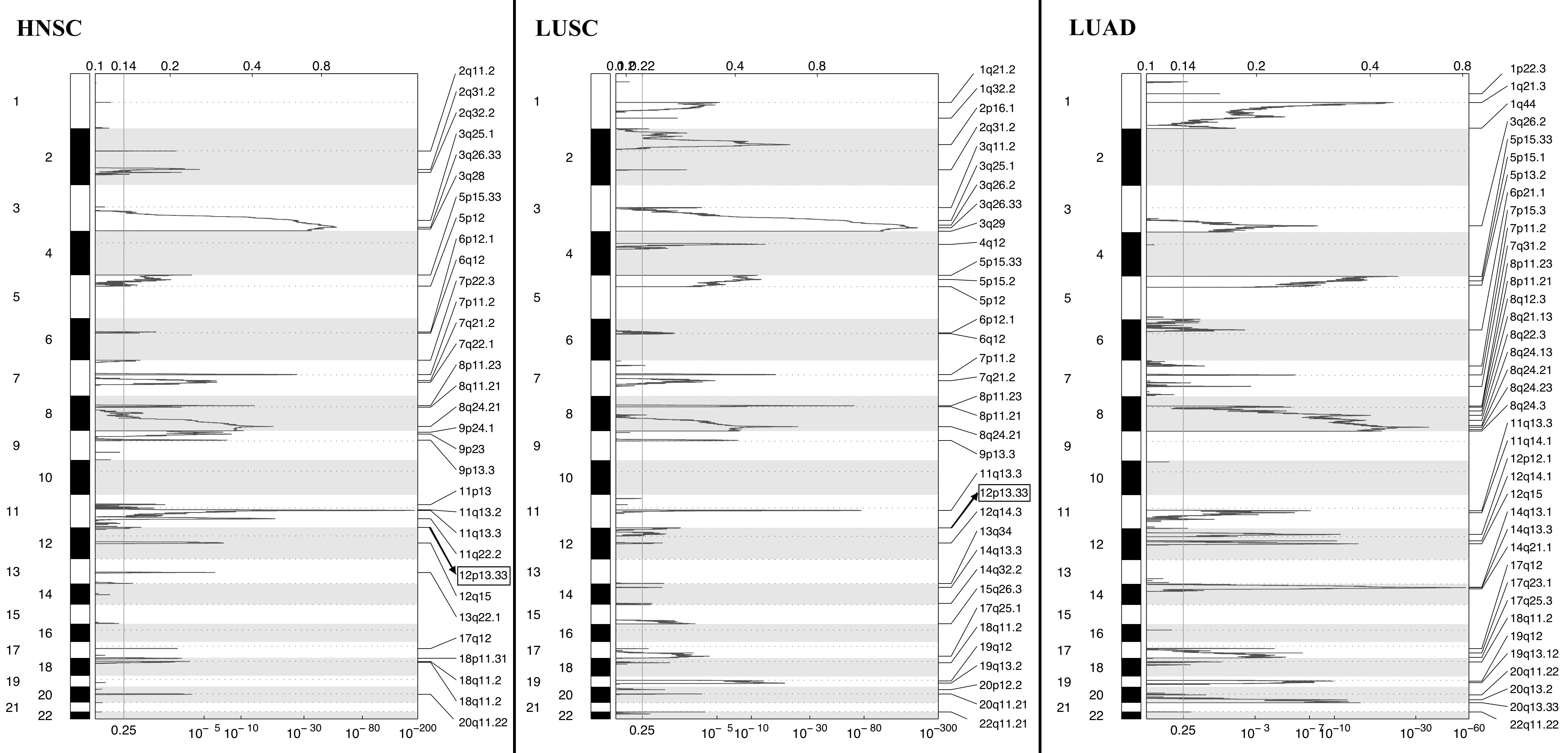


**Table A.** Population stratification sensitivity analysis. Model 1 is the original association analysis logistic regression, adjusted for sex and study specific country of origin. Model 2 further adjusts for population stratification including the 12 significant eigen vectors (as defined by Tracy-Widom statistics) as covariates in the logistic regression.

|  |  |  |  |  |  |  |  |  |
| --- | --- | --- | --- | --- | --- | --- | --- | --- |
|  |  |  | **Model 1** | | | **Model 2** | | |
| **rs10849605 genotype** | **Cases** | **Controls** | **OR** | **95%CI** | **pvalue** | **OR** | **95%CI** | **pvalue** |
| T/T | 382 | 611 | 1 | ref | | 1 | ref | |
| T/C | 930 | 1272 | 1.19 | (1.02- 1.40) | 0.029 | 1.20 | (1.02- 1.40) | 0.029 |
| C/C | 485 | 648 | 1.25 | (1.04- 1.49) | 0.017 | 1.23 | (1.02- 1.47) | 0.027 |
| OR Trend |  |  | 1.11 | (1.02- 1.22) | 0.019 | 1.10 | (1.01- 1.21) | 0.032 |

**Table B**. eQTL analyses using adjusted and non-adjusted linear models to measure the impact of the rs10849605 genotype on *RAD52* tumor expression levels. The model measures the effect of the protective allele T for rs10849605. Number of individuals taken into account in the model, beta estimates and pvalue are given for the three different cancer types and using the following linear models: 1) Non-adjusted, how the genotype influences the gene expression. 2) For HNSC cancer, the subtype (oral cavity, larynx/hypopharynx or oropharynx) is used as the covariate. 3) *RAD52* somatic copy number is used as the covariate. 4) Since we are interested here in the influence of somatic determinants on an increase of expression and because methylation is inversely correlated with expression (hypermethylated sites tend to decrease expression when hypomethylated sites induce increase in expression), we selected 8 of the 24 CpG sites for being hypomethylated (as defined by a negative M-value across all individuals in all our 3 different cancer sites). Out of these 8, only cg15612927 was significantly associated with expression of *RAD52* in all 3 cancers (pvalue < 0.05). Therefore tumor methylation levels of cg15612927 was used as the covariate. 5) The initial model is adjusted for all three somatic alterations (subtype for HNSC, somatic copy number and methylation levels).

|  |  | **1- Non adjusted** | **2- Adjusted for subtype** | **3- Adjusted for Copy Number** | **4- Adjustment for methylation** | **5- Adjusted for all somatic events** |
| --- | --- | --- | --- | --- | --- | --- |
| **LUAD** | Number of individuals | 125 |  | 122 | 84 | 82 |
|  | Beta estimate | -0.02 |  | -0.09 | -0.08 | -0.10 |
|  | p-value | 0.75 |  | 0.08 | 0.31 | 0.09 |
| **LUSC** | Number of individuals | 223 |  | 213 | 94 | 90 |
|  | Beta estimate | -0.16 |  | -0.17 | -0.25 | -0.16 |
|  | p-value | 8.85E-04 |  | 1.41E-06 | 1.18E-03 | 2.78E-03 |
| **HNSC** | Number of individuals | 263 | 263 | 250 | 263 | 250 |
|  | Beta estimate | -0.15 | -0.15 | -0.11 | -0.15 | -0.11 |
|  | p-value | 9.92E-04 | 7.55E-04 | 4.35E-04 | 8.84E-04 | 4.89E-04 |

**Table C.** eQTL sensitivity analysis. The linear model measures the effect of rs10849605 genotype on *RAD52* tumor expression levels. The first line presents the results on all TCGA cases we accessed. The second line restricts the analysis on TCGA cases predicted to be of European origin. The last line show the results of the same linear model but adjusted for the statistically significant eigen vectors, as defined by Tracy-Widom (5 for HNSC and LUSC, 8 for LUAD).

|  | **HNSC** | | **LUSC** | | **LUAD** | |
| --- | --- | --- | --- | --- | --- | --- |
|  | **n** | **p** | **n** | **p** | **n** | **p** |
| **Original** | 263 | 9.90E-04 | 223 | 8.90E-04 | 125 | 0.75 |
| **In population of European origin** | 215 | 2.40E-03 | 192 | 1.10E-03 | 113 | 0.58 |
| **Adjusted by informative eigen vectors** | 215 | 1.70E-03 | 192 | 1.50E-03 | 113 | 0.79 |

**Table D*.*** 12p13.33 copy number versus expression and eQTL analysis in HNSC and LUSC. Association analysis between copy number and expression levels for each given gene in the 12p13.33 region (left side of the table, ‘NA’ if no CNV or expression data available). For the significant associations only, we performed an eQTL analysis to check how rs10849605 genotype influences each given gene expression levels (right side of the table). Significant results are highlighted in green (Bonferroni correction for multiple testing).

**HNSC**

|  | *expression of gene G ~ copy number of gene G* | | | *expression of gene G ~ rs10849605 genotype* | | |
| --- | --- | --- | --- | --- | --- | --- |
| **Genes (G)** | **pvalue** | **estimate** | **std error** | **pvalue** | **estimate** | **std error** |
| RAD52 | 3.17401E-48 | 1.372423055 | 0.074633684 | 0.000992024 | -0.151558711 | 0.04550605 |
| TULP3 | 4.54483E-41 | 1.488069387 | 0.091336075 | 0.786387422 | -0.013683383 | 0.05043942 |
| ADIPOR2 | 1.59546E-32 | 1.367957132 | 0.099137067 | 0.202608711 | -0.060512712 | 0.0473731 |
| ITFG2 | 2.37932E-25 | 1.201757502 | 0.103036327 | 0.656899862 | -0.021419815 | 0.0481661 |
| FKBP4 | 5.69289E-24 | 1.531002513 | 0.136177463 | 0.422189645 | -0.050357325 | 0.06264173 |
| FOXM1 | 5.7922E-16 | 1.334264178 | 0.15392278 | 0.312393771 | -0.068016792 | 0.06719892 |
| TSPAN9 | 6.09328E-14 | 1.094330017 | 0.137443235 | 0.635939811 | -0.026990571 | 0.05694949 |
| TEAD4 | 1.15602E-13 | 1.08523341 | 0.138031442 | 0.367842655 | -0.053078971 | 0.05884022 |
| WNK1 | 1.09879E-10 | 0.933534763 | 0.138495863 | 0.896183809 | -0.007898185 | 0.06047115 |
| NRIP2 | 7.0604E-07 | 0.434156547 | 0.085285879 | 0.579275478 | -0.018995509 | 0.03421761 |
| ERC1 | 2.44903E-06 | 0.638473932 | 0.13233025 | 0.915640548 | 0.005886496 | 0.05551763 |
| DCP1B | 0.04792288 | 0.380488755 | 0.191402812 | NA | NA | NA |
| LRTM2 | 0.0522753 | -0.09395543 | 0.048176576 | NA | NA | NA |
| CACNA2D4 | 0.0575311 | 0.147998192 | 0.077562502 | NA | NA | NA |
| CACNA1C | 0.5238151 | 0.071047823 | 0.111293851 | NA | NA | NA |
| AC026369.1 | NA | NA | NA | NA | NA | NA |
| IQSEC3 | NA | NA | NA | NA | NA | NA |
| SLC6A12 | NA | NA | NA | NA | NA | NA |
| SLC6A13 | NA | NA | NA | NA | NA | NA |
| KDM5A | NA | NA | NA | NA | NA | NA |
| CCDC77 | NA | NA | NA | NA | NA | NA |
| B4GALNT3 | NA | NA | NA | NA | NA | NA |
| NINJ2 | NA | NA | NA | NA | NA | NA |
| FBXL14 | NA | NA | NA | NA | NA | NA |
| WNT5B | NA | NA | NA | NA | NA | NA |
| AC005841.1 | NA | NA | NA | NA | NA | NA |
| RHNO1 | NA | NA | NA | NA | NA | NA |

**LUSC**

|  | *expression of gene G ~ copy number of gene G* | | | *expression of gene G ~ rs10849605 genotype* | | |
| --- | --- | --- | --- | --- | --- | --- |
| **Genes (G)** | **pvalue** | **estimate** | **std error** | **pvalue** | **estimate** | **std error** |
| TULP3 | 3.17174E-37 | 1.639019836 | 0.104581629 | 0.648170153 | -0.025504157 | 0.05581633 |
| RAD52 | 2.81834E-29 | 1.250836896 | 0.095042516 | 0.000884502 | -0.157724208 | 0.04679122 |
| ADIPOR2 | 7.56816E-26 | 1.397584256 | 0.115797337 | 0.262432325 | -0.059498087 | 0.05295648 |
| FOXM1 | 6.48506E-25 | 2.121957535 | 0.180289727 | 0.945781881 | 0.005827365 | 0.08559327 |
| ITFG2 | 2.64883E-22 | 1.30310299 | 0.119302197 | 0.453648207 | -0.040664645 | 0.05417094 |
| FKBP4 | 3.38918E-19 | 1.58354483 | 0.16009611 | 0.542639395 | -0.043665956 | 0.07161032 |
| WNK1 | 1.30872E-14 | 1.299455058 | 0.156729147 | 0.758161592 | -0.020645743 | 0.0669713 |
| ERC1 | 5.81142E-14 | 1.22474657 | 0.152060395 | 0.437492829 | -0.048474611 | 0.06231918 |
| TEAD4 | 8.40837E-14 | 1.439620228 | 0.180061793 | 0.414758023 | -0.060689744 | 0.07427564 |
| NRIP2 | 1.93466E-06 | 0.596510163 | 0.121813987 | 0.071839267 | -0.084014811 | 0.04644751 |
| DCP1B | 9.6381E-05 | 0.709013802 | 0.178336822 | 0.139349082 | -0.099909505 | 0.0673442 |
| TSPAN9 | 0.000236891 | 0.722983146 | 0.19329923 | 0.512105312 | -0.048274861 | 0.0735197 |
| CACNA2D4 | 0.02804118 | 0.24652345 | 0.111449538 | NA | NA | NA |
| CACNA1C | 0.3221018 | -0.117177642 | 0.118065893 | NA | NA | NA |
| LRTM2 | 0.3352702 | -0.08568336 | 0.088721223 | NA | NA | NA |
| AC026369.1 | NA | NA | NA | NA | NA | NA |
| IQSEC3 | NA | NA | NA | NA | NA | NA |
| SLC6A12 | NA | NA | NA | NA | NA | NA |
| SLC6A13 | NA | NA | NA | NA | NA | NA |
| KDM5A | NA | NA | NA | NA | NA | NA |
| CCDC77 | NA | NA | NA | NA | NA | NA |
| B4GALNT3 | NA | NA | NA | NA | NA | NA |
| NINJ2 | NA | NA | NA | NA | NA | NA |
| FBXL14 | NA | NA | NA | NA | NA | NA |
| WNT5B | NA | NA | NA | NA | NA | NA |
| AC005841.1 | NA | NA | NA | NA | NA | NA |
| RHNO1 | NA | NA | NA | NA | NA | NA |
